# Topographical and Chemical Inductive Cues Synergistically Enhance the Schwann Cell Differentiation of Aligned Dental Pulp Stem Cell Sheets

**DOI:** 10.1155/2023/7958770

**Published:** 2023-07-18

**Authors:** Michelle D. Drewry, Kristi Rothermund, Fatima N. Syed-Picard

**Affiliations:** ^1^Department of Bioengineering, Swanson School of Engineering, University of Pittsburgh, Pittsburgh, PA, USA; ^2^Center for Craniofacial Regeneration, School of Dental Medicine, University of Pittsburgh, Pittsburgh, PA, USA; ^3^Department of Oral and Craniofacial Sciences, School of Dental Medicine, University of Pittsburgh, Pittsburgh, PA, USA; ^4^McGowan Institute for Regenerative Medicine, Pittsburgh, PA, USA

## Abstract

Peripheral nerves have an inherent capacity for regeneration, but these Schwann cell-mediated mechanisms are insufficient for severe injuries. With current clinical treatments, slow regeneration and aberrant reinnervation result in poor functional outcomes. Dental pulp stem cells (DPSCs) offer a promising source of therapeutic neurotrophic factors (NTFs), growth factors that stimulate axon regeneration. Previously, we established that DPSCs can generate scaffold-free sheets with a linearly aligned extracellular matrix (ECM). These sheets provide trophic cues via the DPSCs and directional cues through the aligned ECM to both accelerate and orient axon outgrowth, thus providing a biomaterial capable of addressing the current clinical challenges. DPSCs have a propensity for differentiating into Schwann cells (SC-DPSCs), further enhancing their endogenous NTF expression. Here, we evaluated the effect of inducing SC differentiation on the neuroregenerative bioactivity of our DPSC sheets. These sheets were formed on substrates with linear microgrooves to direct the cells to deposit an aligned ECM. Inducing differentiation using an SC differentiation medium (SCDM) increased NTF expression 2-fold compared to unaligned uDPSC sheets, and this effect was amplified in linearly oriented SC-DPSC sheets by up to 8-fold. Furthermore, these aligned SC-DPSC sheets remodeled the sheet ECM to more closely emulate a regenerative neural microenvironment, expressing 8-fold and 2 × 10^7^-fold more collagen IV and laminin, respectively, than unaligned uDPSC sheets. These data demonstrate that the chemical cues of the SCDM and the mechanotransductive cues of the aligned cell sheet synergistically enhanced the differentiation of DPSCs into repair SC-like cells. To evaluate their functional effects on neuritogenesis, the DPSC sheets were directly cocultured with neuronally differentiated neuroblastoma SH-SY5Y cells. In this *in vitro* culture system, the aligned SC-DPSC sheets promoted oriented neurite-like outgrowth similar to aligned uninduced DPSC sheets and increased collateral branching, which may emulate stages associated with natural SC-mediated repair processes. Therefore, linearly aligned SC-DPSC sheets have the potential to both promote nerve regeneration and reduce aberrant reinnervation, thus providing a promising biomaterial for applications to improve the treatment of peripheral nerve injury.

## 1. Introduction

Peripheral nerve injuries (PNIs), caused by trauma, disease, or surgery, leave patients with lifelong disabilities that have a significant impact on both physical and mental health. Specifically, an estimated 26% of individuals older than 65 years of age suffer from sustained peripheral neuropathies not associated with a predisposing disorder [1–3]. While peripheral nerves are innately able to regenerate over short distances, these mechanisms are insufficient for longer, more severe segmental defects. The gold standard treatment for such injuries is the use of autograft tissue to bridge the nerve gap. Since autografts are limited by the availability of donor tissue, the mismatch between the graft and injured nerves, and loss of function at the donor site, engineered conduits offer an alternative method for nerve reconstruction [4, 5]. Even with autografts or commercially available conduits, though, regeneration is slow and inefficient, taking up to 19 months [1]. Furthermore, axon misdirection and branching during nerve regrowth cause aberrant reinnervation and thus also contribute to poor clinical outcomes. Therefore, an ideal nerve therapy would be a conduit capable of providing both neurotrophic cues, to enhance and accelerate axon regeneration, and directional cues, to guide regenerating axons.

One cause of limited PNI recovery is diminished Schwann cell (SC) activity [6–9]. SCs are neural crest-derived glial cells that support peripheral nerve maintenance and regeneration, partially through the production of neurotrophic factors (NTFs) [10]. These growth factors, such as brain-derived neurotrophic factor (BDNF), glial-derived neurotrophic factors (GDNF), and neurotrophin-3 (NT-3), act to promote axon regeneration and growth. While SCs produce high levels of NTFs at the early stages of PNI, this expression diminishes with chronic denervation, which is associated with reduced axon regeneration [8]. Preclinical animal studies have shown that this impaired regeneration can be reversed by treating injured nerves with exogenous NTFs, which stimulate axon growth and improve functional outcomes [11–13]. Because of the natural transient expression of NTFs, a continuous supply of these growth factors is needed to produce a meaningful therapeutic effect [14].

Cell therapies offer a means of sustained NTF delivery. With their innate neurotrophic activity, SCs are an attractive option for such treatments, but they are difficult to isolate and maintain in culture, thus limiting their therapeutic potential [10]. Alternatively, dental pulp stem/progenitor cells also endogenously express high levels of NTFs [13, 15]. Located within the central soft, innervated tissue of the tooth, the dental pulp contains a population of stem/progenitor cells embryonically derived from the neural crest, similar to SCs [16]. These dental pulp stem/progenitor cells (DPSCs) produce higher concentrations of NTFs than other mesenchymal stem cells and are capable of promoting nerve regeneration [13, 15, 17]. Because of their accessibility from autologous sources and propensity for culture expansion, DPSCs offer a more feasible source of cells than SCs for PNI therapies. Our research group has previously shown that DPSCs can be formed into scaffold-free cell sheets that act as an NTF delivery system and enhance axon regeneration in a rodent preclinical nerve crush model [17]. Furthermore, DPSCs can be chemically induced to differentiate into SCs [18–22], which has been shown to increase their neurotrophic activity and therefore could further enhance the neuroregenerative effects of these cell sheets.

Beyond their NTF expression, SCs also support nerve regeneration through the formation of bands of Büngner, in which the cells align and provide a physical bridge to orient extending axons. In more severe injuries, SCs cannot extend these bands across the entire nerve gap, thus causing axon misdirection and aberrant reinnervation. Such inaccurate regeneration can clinically manifest as synkinesis, in which the voluntary movement of one muscle results in the involuntary activation of a different muscle [23]. To bypass these challenges, nerve conduits have been engineered to contain linearly aligned ECM-scaled fibers to mimic the bands of Büngner [24]. These topographies are capable of providing directional guidance cues to chaperone regenerating axons and improving functional recovery [25].

We have previously shown that DPSC sheets can be engineered to contain a linearly aligned ECM. These aligned DPSC sheets provided both trophic cues and directional cues capable of inducing oriented neurite outgrowth by neuronal cells [26]. To engineer these linearly aligned cell sheets, DPSCs were cultured on a substrate with linear microgrooves, which provided contact guidance cues that induced the cells to align and deposit a linearly aligned ECM. It has been previously established that such microtopographies can affect stem cell orientation, morphology, and differentiation [27–31]. Specifically, substrate topography can affect the differentiation of mesenchymal stem cells into SCs [32, 33].

The goal of this study was to evaluate how inducing SC differentiation affects the neuroregenerative capacity of scaffold-free DPSC sheets. Here, DPSCs were cultured on either flat or microgrooved substrates and then induced to differentiate into SC-like cells (SC-DPSCs) using an SC differentiation medium (SCDM). The individual and combinatorial effects of both the chemical signals (SCDM) and contact guidance cues (microgrooved substrates) on DPSC sheet structure, composition, and bioactivity were evaluated. These aligned SC-DPSC sheets can be used as a bioactive conduit material capable of both enhancing and directing axon regeneration.

## 2. Materials and Methods

### 2.1. Isolation of DPSCs

DPSCs were isolated from healthy adult human third molars collected at the University of Pittsburgh School of Dental Medicine using similar methods to those previously published [17, 26, 34, 35]. These teeth were extracted for clinical purposes and deidentified prior to transfer to research staff; therefore, this study is not considered human subject research (University of Pittsburgh Institutional Review Board STUDY21040099). Teeth were stored in 1x phosphate-buffered saline (PBS; Gibco) with 1X penicillin and streptomycin (P/S; Gibco). Within 24 hours after the extraction, the pulp was isolated from the teeth, minced, and then enzymatically digested for 1 hour at 37°C in 3 mg/ml collagenase (EMD Millipore) and 4 mg/ml dispase (Worthington Biochemical). After filtering the digested tissue using a 70 *μ*m cell strainer, the isolated DPSCs were cultured in Dulbecco's Modified Eagle Medium (DMEM; Gibco), 20% fetal bovine serum (FBS; Atlanta Biologicals), and 1X P/S. At 80% confluence, the cells were passaged and cryopreserved, using DPSCs from passages 2–5 to engineer the cell sheets.

### 2.2. Formation of DSPC Sheets

DPSCs were cultured on either flat substrates or substrates with linear microgrooves, as previously reported [26]. The linear grooved substrates contained grooves that are 10 *μ*m wide, spaced 10 *μ*m apart, and 10 *μ*m deep. The linear polymer substrates were formed by curing Sylgard 184 polydimethylsiloxane (PDMS) at 9 : 1 ratio with the manufacturer-provided curing agent on a silicon wafer with the negative features; the cured PDMS was then cut to fit the wells of a 12-well plate. Flat polymer substrates were formed by curing PDMS on a flat surface. The PDMS substrates were coated with 2 *μ*g/cm^2^ laminin (Gibco), and then, UV sterilized. DPSCs were plated on the laminin-coated PDMS substrates at a density of approximately 42,000 cells/cm^2^ in growth media containing DMEM, 20% FBS, 1% P/S, 50 *µ*g/ml L-ascorbic acid (Sigma-Aldrich), and 5 ng/ml fibroblast growth factor (FGF; Peprotech) as previously described [17, 26]. The DPSCs were cultured until they formed a robust cell sheet, changing the culture media every 2-3 days. A Nikon ECLIPSE Ti microscope was used to capture phase contrast images of the cell sheets.

### 2.3. Schwann Cell Differentiation of the DPSC Sheets

Following cell sheet formation, a subset of the DPSC sheets was differentiated into SCs using methods similar to those previously described [18, 19, 36–40]. To induce SC differentiation, cell sheets were cultured in serum-free medium containing 1 mM *β*-mercaptoethanol for 24 h, followed by 72 h of culture in growth medium containing 35 ng/ml all-trans-retinoic acid and 50 *μ*g/ml L-ascorbic acid. The medium was then switched to SC differentiation medium (SCDM) comprised of growth media, 5 *μ*M forskolin, 10 ng/ml fibroblast growth factor 2, 5 ng/ml platelet-derived growth factor-AA, 200 ng/ml heregulin *β*-1, and 50 *μ*g/ml L-ascorbic acid. L-Ascorbic acid was included in the retinoic acid medium and the SC differentiation medium to promote ECM assembly and maintenance for the formation of robust cell sheets.

### 2.4. Immunofluorescent Staining of the DPSC Sheets

Cell sheets were washed twice using PBS, then fixed in 10% formalin and permeabilized with 0.1% Triton X-100 (Sigma-Aldrich). Immunohistochemical staining was performed using a primary antibody against type I collagen (Abcam) and secondary antibody Alexa Fluor 488 anti-rabbit IgG (ThermoFisher). In addition, sheets were stained with phalloidin 594 (Abcam) and DAPI (Sigma-Aldrich) to image the actin cytoskeleton and nuclei, respectively. Negative controls were performed by omitting the primary antibodies and staining the tissues with the secondary antibodies only. The Nikon ECLIPSE Ti, ZEISS Scope.A1 AXIO, or Nikon TE 2000 microscopes were used to image the stained cell sheets using z-stacks with a step size of 5 *μ*m, and z-projections were performed using the FIJI (ImageJ) software and the Extended Depth of Field plugin [41].

### 2.5. Quantification of Nuclear Aspect Ratio and DPSC Sheet Alignment

Nuclear aspect ratio (NAR) was quantified using FIJI software. Images of the DAPI stained nuclei were converted to 8-bit grayscale, and a standardized threshold sets to only include the nuclear fluorescent signal. The Analyze Particles function was then used to measure the aspect ratio of the nuclei, which ranged from 0 to 1, with 1 being perfectly circular.

The nuclear, actin filament, and collagen fiber alignment of the cell sheets was evaluated by measuring the angle of the major axis of the feature of interest relative to the angle of the substrate features, as previously described [26]. Using this method, an alignment angle of 0° indicates the alignment of the feature, which is parallel with the substrate topography. For the sheets formed on flat PDMS, an arbitrary substrate feature angle was used as the reference. Using FIJI software, component images were converted to 8-bit grayscale. For the nuclear alignment, the measure function was used to quantify the nuclei angle, and for the actin and collagen orientation, directionality was assessed using the Fourier components plugin. Histograms were created using RStudio and the ggplot2 package [42, 43], with angles ranging from −90° to 90° and separated into 10° bins.

### 2.6. Histological Analysis of the DPSC Sheets

DPSC sheets were washed twice with 1X PBS and fixed in 10% formalin. Then, the cell sheets were processed for standard paraffin embedding and sectioned in cross section at a 5 *µ*m thickness. Sections were either stained with hematoxylin and eosin (H&E), or immunohistochemical staining. The immunohistochemical staining was performed using a primary antibody against type I collagen (Invitrogen), type IV collagen (Proteintech), laminin (Novus Biologicals), SOX10 (Sigma-Aldrich), BDNF (ThermoFisher), GNDF (ThermoFisher), or NT-3 (ThermoFisher) and secondary antibody Alexa Fluor 488 anti-rabbit IgG (ThermoFisher), Alexa Fluor 596 anti-rabbit IgG (ThermoFisher), Alexa Fluor 488 anti-mouse IgG (ThermoFisher), or Alexa Fluor 596 anti-mouse IgG (ThermoFisher). The Nikon ECLIPSE Ti, ZEISS Scope.A1 AXIO, or Nikon TE 2000 microscopes were used to capture images. ImageJ was used to process the images and quantify the relative protein intensities. The reported protein intensities (*n* = 3) were calculated by averaging the fluorescent intensities from three areas of a single cell sheet. These values were then normalized to the number of cells within the field of view.

### 2.7. Quantitative Real-Time Polymerase Chain Reaction (qPCR)

The QIAGEN QIAshredder and RNeasy Mini kits were used to isolate DPSC sheet RNA, per the manufacturer's instructions, and a NanoDrop instrument (Thermo Scientific) was used to measure RNA concentration. qPCR was performed with the TaqMan RNA to *C*_*t*_ One Step Kit (Applied Biosystems) using human *BDNF*, *GDNF*, *NT-*3, *COL1A1*, *COL4A1*, *LAMA2*, and housekeeping gene 18*-S* primers (TaqMan Gene Expression Assays, Applied Biosystems). The relative gene expressions were calculated using the 2^−ΔΔCt^ method, comparing the expression from uninduced DPSC sheets and SC-differentiated DPSCs sheets formed on the flat and microgrooved substrates to the flat uninduced DPSC sheets. The data represent the averages of technical triplicates.

### 2.8. Assessment of Neurite Outgrowth In Vitro

Neuroblastoma cells (SH-SY5Y; ATCC) were cultured in a growth medium containing DMEM/F12 (Gibco) and 10%FBS. Once they reached 50% confluency, the cells were induced to differentiate into a neuronal lineage using a media of DMEM, 1% FBS, and 10 µM all-trans-retinoic acid (RA; Acros Organics) [17, 26, 44, 45]. The RA-treated SH-SY5Y cells were then cocultured with the DPSC sheets at a density of 8.6 × 10^3^ cells/cm^2^, as previously reported [26]. After 4 days, the cocultures were washed 3x with PBS and fixed in 10% formalin. It was then permeabilized using 0.1% Triton 100-X. Immunofluorescent staining was performed using primary antibodies against neurofilament 200 (NF200) (Novus Biologicals) and secondary antibody Alexa Fluor 546 anti-rabbit IgG (ThermoFisher). Nuclei were stained using DAPI. Z-stack images were captured with a step size of 5 *μ*m using Nikon ECLIPSE Ti, ZEISS Scope.A1 AXIO, or Nikon TE 2000 microscopes. Images were deconvoluted using the NIS-Elements software (Nikon) and z-projected using FIJI software and the Extended Depth of Field plugin [41].

The outgrowth of neurite-like projections was evaluated by quantifying the neurite-like projection alignment, net displacement, total length, and branching, using previously established methods [26]. A neurite-like structure was defined as a projection longer than twice the length of the soma. The orientation of the neurite-like processes was measured by comparing the angle of the cell process to that of the substrate feature, similar to the previously described alignment methods. Net displacement, the net length of the cell projections from the soma to the end, and the total length were quantified using FIJI software. The alignment, net displacement, and total length plots were made using RStudio and the ggplot2 package [42, 43]. The branching of neurite-like processes was measured by counting the total number of branches per projection and the number of neurite-like projections with branches normalized to the total number of projections evaluated. In total, greater than 250 neurite-like projections were assessed per group.

### 2.9. Statistical Analyses

Statistical analyses were performed using RStudio, and the data are represented as the mean ± the standard deviation of the mean [42]. For the NAR, PCR, IHC intensity, and neurite-like length evaluations, statistical analyses were performed using the ANOVA and Tukey's posthoc statistical tests. With the alignment measurements, the two-sample Kolmogorov–Smirnov test was used to assess statistical differences between the groups.

## 3. Results

### 3.1. DPSC Sheet Formation and Characterization of Cell and ECM Alignment

**Figure 1:**
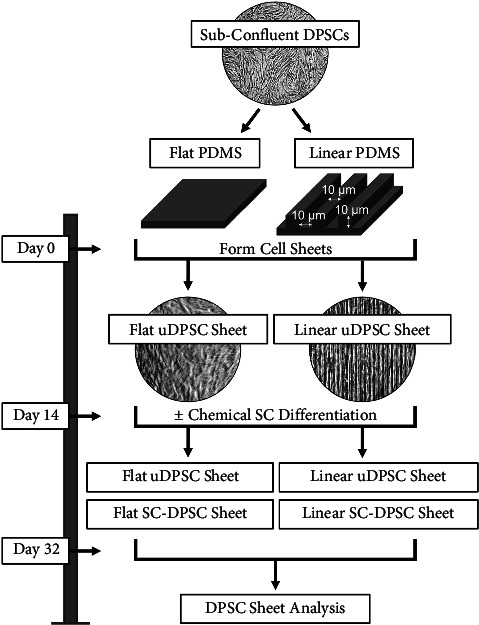
Overview of the dental pulp stem cell (DPSC) sheet formation. Subconfluent DPSCs were cultured on either a flat substrate or a substrate with linear microgrooves, forming flat uninduced DPSC (uDPSC) sheets or linear uDPSC sheets. A subset of these sheets was chemically differentiated into flat or linear Schwann cell-induced DPSC (SC-DPSC) sheets, after which all sheets were collected and used for subsequent analyses.

DPSC sheets were formed on either microgrooved or control flat substrates, as previously reported [26]. Following cell sheet formation, a subset of the DPSC sheets was chemically induced to differentiate into SCs using SC differentiation medium (SCDM), as seen in Figure 1 [18, 19]. Therefore, four experimental groups were included in this study: uninduced DPSC (uDPSC) sheets formed on flat or linear substrates and SC-induced DPSC (SC-DPSC) sheets formed on flat or linear substrates.

**Figure 2:**
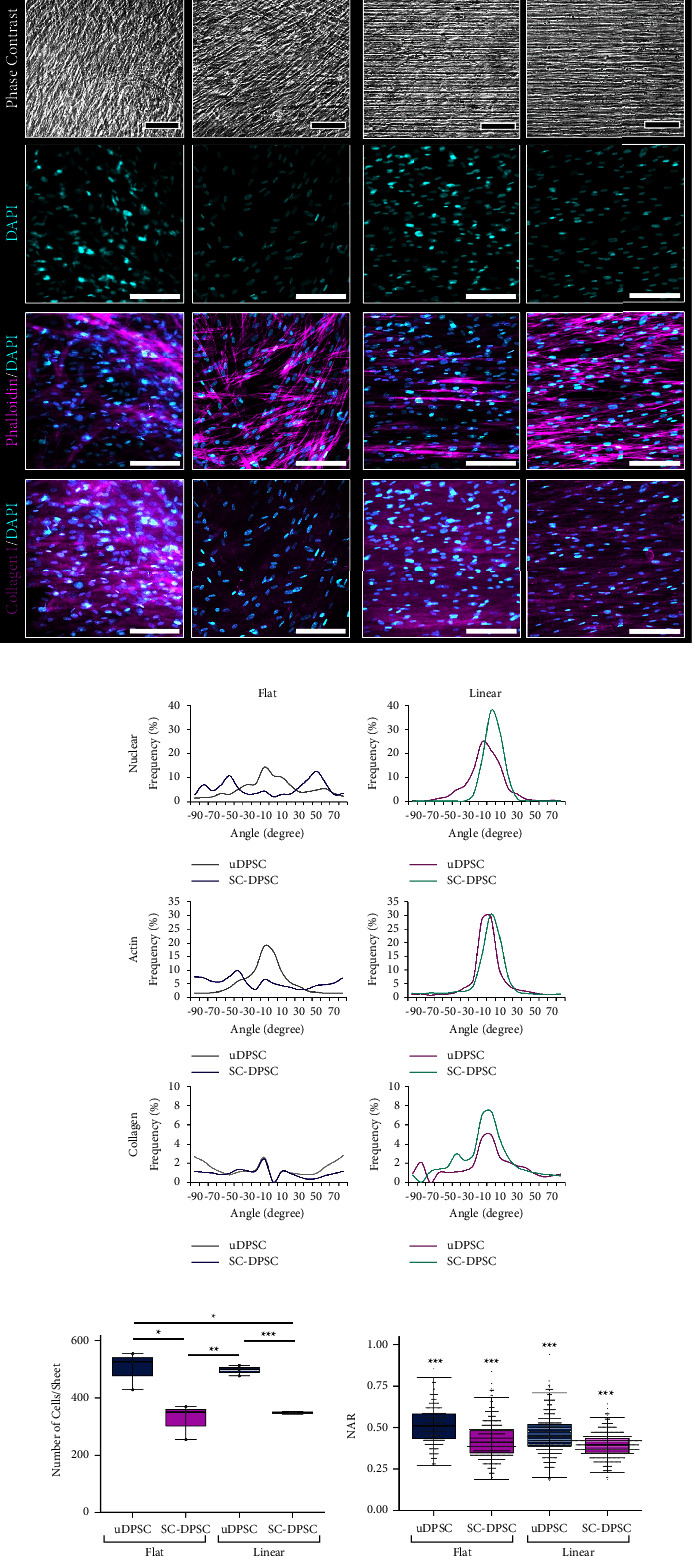
Structural characterization of the Schwann cell-induced dental pulp stem cell (SC-DPSC) sheets formed on the microgrooved or flat substrates. (a) Phase contrast images, DAPI staining (cyan), phalloidin staining (magenta), and immunostaining against type I collagen (magenta) indicated that DPSCs cultured on the linear topography oriented in parallel with the underlying microgrooves and deposited a linearly aligned extracellular matrix (ECM). (b) Nuclei, actin cytoskeleton, and collagen fiber alignment were quantified relative to the underlying substrate, with an angle closer to 0° indicating alignment of the feature parallel to the substrate topography. These data indicated alignment on the linear topography but not the flat topography. Statistical analysis of nuclear alignment: uninduced DPSC (uDPSC)-flat vs uDPSC-linear (*p*=0.022), SC-DPSC-flat vs SC-DPSC-linear (*p* < 0.001), SC-DPSC-linear vs uDPSC-linear (*p*=0.0077). Statistical analysis of actin alignment: uDPSC-flat vs uDPSC-linear (*p*=0.021), SC-DPSC-flat vs SC-DPSC-linear (*p* < 0.001), SC-DPSC-flat vs uDPSC-flat (*p*=0.021). Statistical analysis of collagen alignment: SC-DPSC-flat vs SC-DPSC-linear (*p*=0.022). (c) Using the DAPI images to count the number of cells per sheet showed that the uDPSC sheets contained significantly more cells than the SC-DPSC sheets. (d) Further analysis of the DAPI images indicated that the linear SC-DPSCs had the smallest nuclear aspect ratio (NAR) and were thus more elongated than the other DPSCs, with positive error bars representing standard deviation and negative error bars standard error of mean. (^*∗*^: *p* value <0.05, ^*∗∗*^: *p* value <0.01, ^*∗∗∗*^: *p* value <0.001). Scale bars: (a) phase contrast = 100 *μ*m, fluorescence = 150 *μ*m.

Phase contrast imaging showed DPSC sheets formed in all culture conditions that were solid and cellular (Figure 2(a)). The SC-DPSCs appeared to have more elongated, bipolar cell morphology. Quantifying their nuclear alignment showed that DPSCs cultured on the linear substrates had significantly more aligned nuclei than those formed on the flat substrates (Figure 2(b)). Additionally, of the DPSCs cultured on the linear microtopography, the SC-DPSCs had more aligned nuclei than the uDPSCs. This difference was not observed between the uDPSCs and SC-DPSCs on the flat substrate. As demonstrated in Figures 2(a) and 2(b), phalloidin staining similarly indicated that both the SC-DPSCs and uDPSCs cultured on the linear microtopography contained a linearly aligned actin cytoskeleton, and this alignment was not present in DPSCs that formed sheets on the flat substrate. Unlike the nuclear alignment, there was no significant difference in actin organization between the uDPSCs and SC-DPSCs formed on the linear substrates. Conversely, the actin cytoskeleton comprising SC-DPSCs cultured on the flat substrates was more aligned that that of the uDPSCs. These fibers, though, appear less dense in the SC-DPSC sheets compared to the uDPSC sheets. Likewise, immunostaining against type I collagen, a structural component of the ECM, indicated that the DPSCs cultured on the grooved substrates deposited a more linearly aligned ECM than those on the flat substrates, and this expression was similarly less dense in the SC-DPSC sheets (Figures 2(a) and 2(b)).

Quantitative image analysis of the DAPI images like those shown in Figure 2(a) showed that the SC-DPSC sheets contained significantly less cells than the uDPSC sheets (Figures 2(c)). These images were also used to quantify the nuclear aspect ratio (NAR), with values closer to 1 indicating a more circular nucleus (Figures 2(d)). SC-DPSCs had more elongated nuclei than uDPSCs, and the nuclei of linearly aligned DPSCs also had significantly greater elongation than their unaligned counterparts. Thus, the linearly aligned SC-DPSC sheets contained fewer cells than the other DPSC sheets, and these cells contained more elongated nuclei.

### 3.2. Effects of Substrate Topography and the Induction of SC Differentiation on DPSC Sheet Structure and Composition

**Figure 3:**
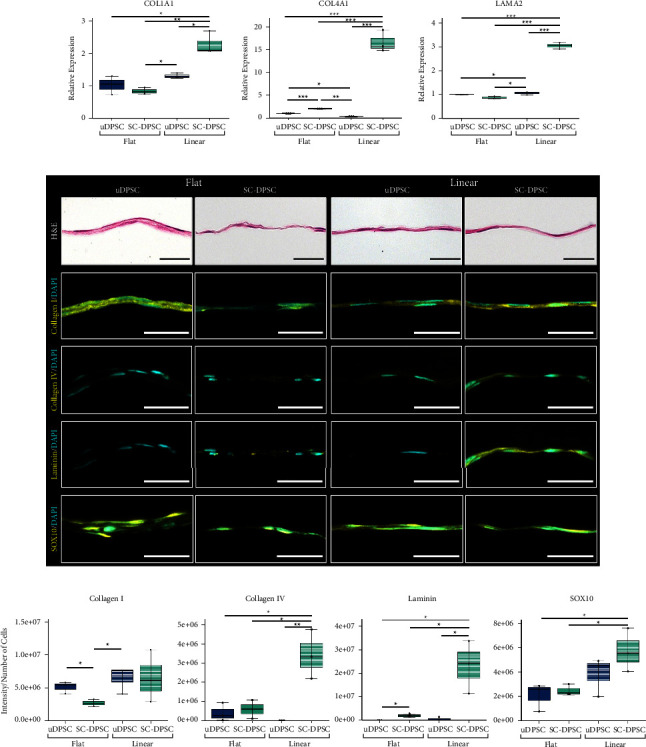
Extracellular matrix (ECM) composition of the dental pulp stem cell (DPSC) sheets. (a) Analysis of the mRNA expression of *COL1A1*, *COL4A1*, and *LAMA2* showed an increased concentration of these ECM genes in the linearly aligned Schwann cell-induced DPSC (SC-DPSC) sheets. (b) H&E staining of histological cross sections of the DPSC sheets showed that all sheets were solid and cellular, yet those cultured on the linear topography or in SC differentiation medum (SCDM) were thinner than the flat uninduced DPSC (uDPSC) sheets. Immunostaining against collagen (I) collagen IV, and laminin (yellow) demonstrated that the linear SC-DPSCs expressed more of these ECM proteins, as well as SC marker SOX10. (c) Quantifying this expression as the total intensity normalized to the number of cells validated that the linear SC-DPSC sheets contained more of the basement membrane proteins and SC marker SOX10 than the other DPSC sheets. (^*∗*^: *p* value <0.05, ^*∗∗*^: *p* value <0.01, ^*∗∗∗*^: *p* value <0.001). Scale bars = 50 *μ*m.

The effects of inducing SC differentiation on the ECM composition of the DPSC sheets were characterized by evaluating changes in the expression of type 1 collagen, a common ECM component of connective tissues, and type IV collagen and laminin, which are basal lamina proteins naturally expressed by SCs. The mRNA expression of *COL1A*1, *COL4A1*, and *LAMA2* by the DPSCs was measured using PCR (Figure 3(a)). The aligned SC-DPSCs expressed the greatest levels of *COL1A1*, *COL4A1*, and *LAMA2*. In contrast, the SCDM stimulated the unaligned SC-DPSCs to express less *COL1A1* than their unaligned uDPSC counterparts although they expressed significantly more *COL4A1* than the unaligned uDPSCs.

H&E stained histological cross sections of the cell sheets demonstrated that in all of the experimental conditions, the DPSCs formed solid tissues with multiple cell layers (Figure 3(b)). Interestingly, the H&E staining also indicated that the DPSC sheets engineered either on microgrooved substrates or in SCDM qualitatively appeared thinner than the uDPSC formed on flat substrates. Immunostaining of the histological cross sections confirmed the presence of type I collagen, type IV collagen, and laminin throughout the thickness of the tissue (Figure 3(b)). The fluorescent signal intensity normalized to the number of cells showed that the unaligned SC-DPSCs expressed significantly less collagen I protein compared to the uDPSCs, corresponding with what was observed for *COL1A1* mRNA expression (Figures 3(b) and 3(c)). Unlike the *COL1A1* gene expression, though, there was no significant difference between the amounts of collagen I protein produced by the aligned SC-DPSCs compared to the other experimental groups. As for the collagen IV and laminin, the protein expression followed a similar trend to that of mRNA expression, with the aligned SC-DPSCs producing the greatest levels of collagen IV and laminin protein. In addition, the unaligned SC-DPSCs expressed significantly more laminin than the unaligned uDPSCs.

Immunostaining was also used to detect the expression of the SC marker, SOX10 [46]. SOX10 was expressed by the DPSCs in all experimental conditions (Figure 3(b)), indicating that even uDPSCs express this SC-related protein. Interestingly, quantification of SOX10 expression indicated that aligned SC-DPSCs had the highest expression of this marker. Overall, both SCDM and surface topography caused the aligned SC-DPSCs to express greater SC-related markers and basal lamina proteins than the other DPSC sheets.

### 3.3. Effects of Substrate Topography and SC Differentiation on NTF Expression

**Figure 4:**
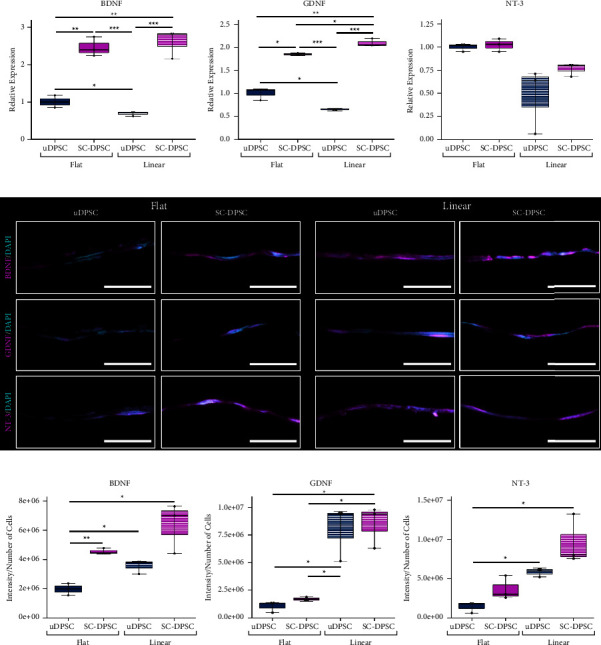
Neurotrophic factor (NTF) expression by dental pulp stem cell (DPSC) sheets. (a) Analysis of NTF mRNA expression indicated that the linear Schwann cell-induced DPSCs (SC-DPSCs) produced greater levels of *BDNF* and *GDNF*. (b) Immunostaining of histological cross sections of the cell sheets showed NTFs protein expression (magenta) localized to the cell sheets in all experimental conditions. (c) Quantifying this expression as the total intensity normalized to the number of cells showed increased NTF expression in the linear uninduced DSPC (uDPSC) sheets and linear SC-DPSC sheets compared to the flat uDPSC sheet. (c) Taking the total intensity normalized to the number of cells validated this increase in expression, especially in the linearly aligned SC-DPSC sheets. (^*∗*^: *p* value <0.05, ^*∗∗*^: *p* value <0.01, ^*∗∗∗*^: *p* value <0.001). Scale bars: (b) fluorescence = 50 *μ*m.

The mRNA expression of NTFs *BDNF*, *GDNF*, and *NT-3* was measured to evaluate the effect of SCDM and linear alignment on DPSC sheet bioactivity (Figure 4(a)). The SC-DPSCs overall produced significantly more *BDNF* and *GDNF* than the uDPSCs, with the linearly aligned SC-DPSCs expressing the highest levels of these genes. In contrast, there was no significant difference in the *NT-3* mRNA expression between the different culture conditions.

To assess the NTF protein expression, cross sections of the DPSC sheets were immunostained to detect BDNF, GDNF, and NT-3, and the protein expressions were quantified by normalizing the total intensity to the number of cells (Figures 4(b) and 4(c)). With the gene expression, the linearly aligned SC-DPSCs produced more NTFs than the other DPSCs though this trend was not always statistically significant. Unaligned uDPSCs expressed the lowest levels of BDNF, GDNF, and NT-3. Both the aligned uDPSCs and aligned SC-DPSCs produced more GDNF than the flat SC-DPSCs. Overall, both gene and protein expression analyses showed that aligned SC-DPSCs produce the greatest levels of NTFs, indicating a synergistic effect between the chemical SCDM inductive and mechanotransductive cues on the DPSC sheet neurotrophic bioactivity.

### 3.4. Functional Effect of the DPSC Sheets on Neuritogenesis

**Figure 5:**
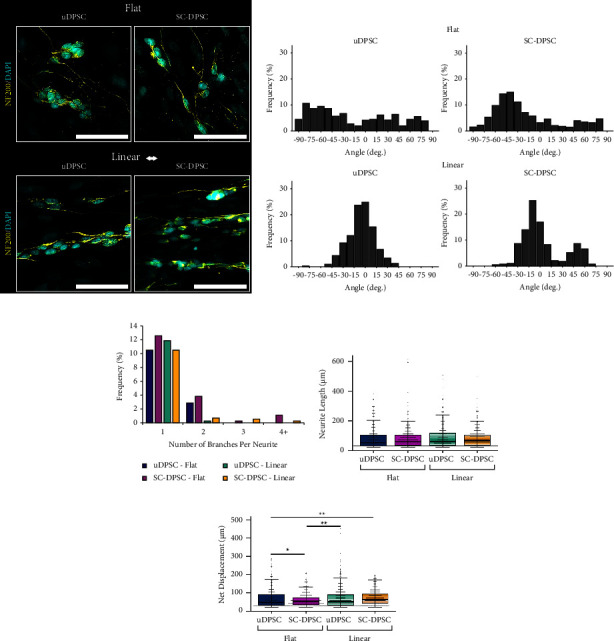
Functional effect of the dental pulp stem cell (DPSC) sheets cocultured with neuronally differentiated neuroblastoma cells. (a) Immunostaining against NF200 (yellow) showed that the neurite-like outgrowth was linearly aligned on the aligned DPSC sheets. (b) Quantifying the alignment of the neurite-like processes validated that more neurite-like processes were aligned on the linear DPSC sheets compared to the flat DPSC sheets; neurite alignment was significantly different among all experimental groups (*p* < 0.001). (c) Schwann cell-induced DPSC (SC-DPSC) sheets induced an increased number of branches per neurite-like process compared to the uninduced DPSC (uDPSC) sheets. (d) A difference in overall length of the neurite-like processes was not detected between the neuronal cells cultured on the various DPSC sheets. (e) In contrast, the net displacement of the neurite-like processes produced by the neuronal cells cultured on the unaligned SC-DPSC sheets was significantly shorter than those on the other DPSC sheets. (^*∗*^: *p* value <0.05, ^*∗∗*^: *p* value <0.01). Scale bars = 100 *μ*m.

To functionally investigate their neuroregenerative capacity, the ability of the DPSC sheets to induce oriented neurite outgrowth was evaluated by coculturing neuronally differentiated SH-SY5Y neuroblastoma cells directly on the cell sheets. All DPSC sheets were able to induce the outgrowth of neurite-like structures from the neuronal cells (Figure 5(a)). Only 10.6% of these neurite-like processes were aligned on the unorganized uDPSC sheets and 13.5% on the unaligned SC-DPSC sheets. However, the linearly aligned uDPSC and SC-DPSC sheets, respectively, induced 63.0% and 50.5% of the neurite-like processes to align linearly (±10°) (Figure 5(b)), supporting that the aligned DPSC sheets could effectively orient extension. Furthermore, although most neurite-like processes can be seen extending linearly on the aligned SC-DPSC sheets, a second set of processes was also observed extending at about 45°. Statistical comparisons across the alignment profiles showed that the alignment of neurite-like processes was significantly different between all groups (*p* < 0.001). Furthermore, the neurite-like processes extending on the linearly aligned cell sheets contained less branches than those on the unaligned sheets. Quantifying the number of branches per neurite-like process showed that the unaligned SC-DPSC sheets induced the greatest number of branches, with 17.8% of the processes containing one or more branches (Figure 5(c)). Comparatively, 13.4% of the neurite-like processes on the unaligned uDPSC sheets contained branches, and only 12.1% and 12.0% were branched on the aligned uDPSC and SC-DPSC sheets, respectively. Moreover, for both the aligned and unaligned culture conditions, only neuronal cells cultured on SC-DPSC sheets contained a subset of the neurite-like processes with 3 or more branch points (Figure 5(c)). Despite the differences in branching, there was no significant difference in total length of the neurite-like processes among the various experimental conditions (Figure 5(d)). When assessing the net displacement of these processes, though, the aligned SC-DPSC sheets induced significantly longer net outgrowth compared to the flat uDPSC sheets (Figure 5(e)). Thus, the aligned DPSC sheets were able to induce oriented neurite-like outgrowth from neuronally differentiated neuroblastoma cells, and inducing SC differentiation did not affect neurite length and displacement but did cause increased neurite branching.

## 4. Discussion

We previously established that uDPSC sheets generated with an aligned ECM elicit neuritogenesis and orient extension [26]. Here, we have built on this prior work and found that stimulating SC differentiation increases the neurotropic bioactivity of these sheets and causes major ECM remodeling. SC-DPSC sheets expressed elevated levels of NTFs and basal lamina proteins compared to their uninduced counterparts supporting that SCDM effectively stimulated the DPSCs to differentiate towards an SC-like phenotype. Moreover, inducing SC differentiation in the DPSC sheets formed on microgrooved substrates further perpetuated these effects and increased the expression of the SC marker SOX10. This indicates that the chemical induction cues supplied by the SCDM, and the mechanotransductive contact guidance cues of the linear cell sheets synergistically enhanced SC differentiation by the DPSCs. This study provides new insight into DPSC biology by highlighting the multiple signaling modalities, soluble, and mechanotransductive that can influence DPSC differentiation towards SC-like cells. Furthermore, the outcomes of this study resulted in the development of a bioactive material containing SC-like cells in a neuro-permissive microenvironment that can be used to treat PNIs.

SC differentiation was induced following cell sheet formation. By inducing SC differentiation after cell sheet formation, the differentiating DPSCs received contact guidance cues from both the underlying microgrooved PDMS substrate and reciprocal cues from the previously DPSC-deposited aligned ECM. These contact guidance cues, as well as the chemical differentiation cues supplied by the SCDM, were able to further stimulate cell and ECM alignment, highlighted by the elevated nuclear alignment in the linear SC-DPSC sheets and actin cytoskeleton alignment in the flat SC-DPSC sheets compared to their uDPSC counterparts. Prior studies have shown that primary and stem cell-derived SCs align with micro- and nano-grooved substrates [33, 38, 47–57], and aligned primary SCs are capable of depositing a linearly aligned ECM in culture [56]. Similarly, here, the contact guidance and chemical SCDM induction cues synergistically enhanced alignment of the cell sheets, creating robust SC-DPSC sheets with the aligned topography needed to direct axon regeneration.

SCs play a key role in remodeling the ECM of nerves both during development and after injury [58–61]. Such changes in the neural ECM, which results in deposition of an aligned basal lamina, are necessary for creating a permissive microenvironment for nerve regeneration. The ECM of the linearly aligned SC-DPSC sheets contained a higher concentration of basal lamina proteins compared to the other DPSC sheets. Additionally, the flat SC-DPSC sheets contained less collagen I than their uDPSC and linear complements. Together, these results show that chemical SC differentiation and contact guidance both directed ECM remodeling of the DPSC sheets. To our knowledge, this is the first report on the synergistic effect of SCDM and substrate topography on ECM reorganization. Separately, though, previous studies have shown that either contact guidance cues or SCDM individually can affect ECM remodeling. For example, the alignment of primary SCs on a substrate composed of microgrooves or electrospun nanofibers; thus, a similar microenvironment in our linearly aligned DPSC sheets resulted in decreased ECM expression [56]. Additionally, cell alignment can promote changes in matrix metalloproteinase (MMP), tissue inhibitor of metalloproteinase (TIMP), and collagen expression [62–65]. Such effects of topography on ECM remodeling, though, are variable depending on the specific cell type and topographical features. It has also been reported that chemical SC differentiation of unaligned uDPSCs caused increased laminin expression [18]. Also, the addition of ascorbic acid to SCDM may have also contributed to the expression of basal lamina proteins by SC-DPSCs. In culture, primary SCs do not synthesize collagen IV or a basal lamina without the addition of ascorbic acid or coculture with neurons. This basal lamina is necessary for robust myelination [59, 66–69]. In this study, ascorbic acid was added to the differentiation medium to promote robust cell sheet formation. This addition likely promoted the production of collagen IV by the SC-DPSCs, and this basal lamina protein expression was further increased in the linearly aligned SC-DPSCs. Therefore, this study uniquely showed that the soluble SCDM cues together with mechanotransductive contact guidance cues synergistically induced ECM remodeling of the DPSC forming a biomimetic microenvironment more conducive to nerve regeneration.

The SC-DPSCs derived from the linearly aligned DPSC sheets adopt an elongated, bipolar morphology resembling natural regenerative SCs [33, 51, 53]. A similar cell morphology has been observed in SC-like cells that are derived from bone marrow or adipose-derived stem cells [36, 37, 70–72]. Quantification of the DPSC NAR revealed that the contact guidance mechanotransduction cues and the SCDM chemical cues synergistically promoted nuclear elongation [73, 74]. Changes in NAR have been associated with changes in gene expression and stem cell differentiation, and culturing cells on linear topographies can induce nuclei elongation and correspondingly affect stem cell differentiation [75, 76].

Interestingly, the scale of topographical cues dictates the lineage of differentiating stem cells. Aligned nanoscaled topographies have been shown to promote the differentiation of bone marrow and adipose-derived stem cells towards an SC phenotype [32, 33]. In contrast, an aligned microscaled topography, similar to the scale of the PDMS substrates used in this study, has been shown to have no effect on SC differentiation [47], but the aligned SC-DPSC sheets exhibited greater SC phenotype than unaligned SC-DPSCs. Here, the DPSCs formed sheets with an aligned ECM prior to the induction of SC differentiation with SCDM. This aligned ECM would provide a nanoscaled topography to the differentiating DPSC and likely played a larger role in promoting SC differentiation than the underlying topography of the microgrooved PDMS. These contact guidance cues in combination with the chemical SCDM induction cues resulted in cells that more closely resembled the morphology of mature and regenerative SCs.

SC differentiation and linear alignment not only affected the ECM remodeling and cell morphology but also independently and synergistically promoted increased NTF expression. Primary SCs innately express high levels of NTFs [77], and prior studies have shown that inducing SC differentiation of bone marrow stem cells, adipose-derived stem cells, and DPSCs can elevate their NTF expression [18, 22, 38, 39, 78]. However, the effects of inducing cell alignment on NTF expression have not been consistently reported where some have shown that alignment of primary or stem cell-derived SCs on a grooved substrate can further increase NTF production [38, 55, 79] while other studies showed that it either had no effect or decreased NTF expression [49, 79–81]. Here, we have demonstrated that linear microgrooves can prompt increased NTF secretion by DPSCs. This expression was further elevated by inducing SC differentiation. We have previously shown that when wrapped around a facial nerve crush injury in rats, unaligned uDPSC sheets improved functional outcomes relative to untreated controls; however, the repaired nerves were not functioning at equivalent levels as healthy nerves [17]. Here, through the synergistic effects of soluble SCDM cues and contact guidance cues, we are enhancing the neurotrophic bioactivity of these DPSCs, which could potentially further improve the effectiveness of the DPSC sheets when utilized in therapeutic applications.

With their high NTF expression and linearly aligned ECM, the aligned SC-DPSC sheets were able to induce oriented neurite-like outgrowth in directly cocultured neuronal cells, but the length of this outgrowth was not substantially different among the different DPSC sheets groups. In contrast, other studies have shown that SCs derived from bone marrow and adipose-derived stem cells promoted increased neurite outgrowth compared to the uninduced stem cells [37–40, 82, 83]. DPSCs innately express higher levels of NTFs than other MSCs [13, 15], and stem cell-derived SCs have been shown to produce more BDNF and GDNF than primary SCs [39, 78]. We have previously confirmed that the uDPSC sheet conditioned media induce neuritogenesis in neuronal cells through primarily the presence of NTFs [17]; here, we could be observing a saturation point in the effect of the NTFs. This would explain the similar neurite-like process lengths on the uDPSC and SC-DPSC sheets despite differences in NTF expression. The in vitro coculture system utilized here confirmed that both the aligned uDPSC and the SC-DPSC sheets could effectively induce and orient neurite-like extension in neuronal cells. However, this in vitro model system does not fully recapitulate the in vivo injury environment. Additionally, in vivo experiments are needed to further compare the functional neuroregenerative effects of these bioactive materials; the increased NTF expression by the SC-DPSCs could be effective in increasing the functional repair in an in vivo injury model.

Both aligned uDPSC and SC-DPSC sheets effectively oriented neurite-like outgrowth by neuronally differentiated neuroblastoma cells in culture; however, a subset of neurite-like processes extends on the aligned SC-DPSC sheets deterred at approximately 45° angle and SC-DPSCs induced increased branching. The effects of axon branching on nerve repair processes are currently debated in the literature, with some reports suggesting that increased branching is associated with axonal misdirection while others postulate that collateral branching could be critical for preferential motor reinnervation [84, 85]. Specifically, one study found no improvement in functional recovery with reduced collateral axon branching [84]. The increased branching observed in the SC-DPSC sheets could be a consequence of the elevated laminin expression since laminin is known to promote neurite branching [58]. Additionally, the reduced type I collagen expression could contribute to 45° realignment of extending neurite-like processes since it has been reported neurites grow along collagen fibers [86]; the extending neurite-like processes could therefore potentially be jumping between different sparse collagen regions of the cell sheet. During natural nerve regeneration processes, primary SCs induce branching during regrowth and then help prune the misdirected collateral branches [87]. Therefore, the SC-DPSC sheets may still promote more accurate reinnervation within *in vivo* nerve injury sites because of their role in selectivity, despite their propensity for inducing increased branching.

Beyond developing a therapy for PNIs, the linearly aligned SC-DPSC sheets may also provide a model system to study SC behavior and the nerve microenvironment. In developing nerves, neural crest progenitor cells differentiate into SC precursor cells that actively produce a fibrillar collagen matrix consisting of type I collagen [59, 88, 89], similar to that of uDPSC sheets generated here. With maturation, natural SCs then begin adopting a more elongated, bipolar morphology and start producing an ECM more reminiscent of a basal lamina, which is necessary for the radial sorting of axons and myelination [58, 59, 88–91]. Mature SCs then decrease their laminin and NTF expression after nerve development [59, 77], but nerve injury pushes these cells to differentiate towards a regenerative phenotype that is characterized by a significant increase in cell elongation and increased NTF and laminin expression [77, 92]. This progression of cell morphology changes, ECM remodeling, and temporal NTF expression in natural nerve development and repair processes is mirrored to those observed when inducing SC differentiation in the aligned DPSC sheets. Prior to SC differentiation, the uDPSC sheets initially mimic the fibrillar collagen microenvironment of an immature nerve. Through the synergistic effect of the linear topography and chemical induction media, these neural crest-derived progenitor cells remodel their ECM, elongate, and express increased levels of NTFs, thus emulating the development and repair processes of natural SCs.

By forming cell sheets composed of neurotrophic DPSCs with their aligned, endogenous ECM, we have engineered a bioactive material capable of both promoting and guiding axon regeneration. The contact guidance cues from linearly aligned topographies and the soluble chemical cues from an established SCDM synergistically promote the differentiation of DPSCs into SC-like cells. The resulting aligned SC-DPSC sheets contained a neurotrophic microenvironment that mimics that of native regenerative nerve tissue. This biomaterial can be used for numerous therapeutic applications, such as a biological wraps to improve repair of damaged nerves treated with the current standard of care or as materials to generate conduits to bridge severe gap injuries. Additionally, the use of these cell sheets is not limited solely to PNIs: these sheets could also be applied to enhance repair of other nerve injuries, including those of the spinal cord or the optic nerve. These aligned SC-DPSC sheets could also be used as experimental models to study the biological processes involved in SC differentiation, neurite-SC interactions, or neurite-ECM interactions. Moreover, further complexity could be introduced to this model system by the addition of other support cells involved in nerve repair.

## Data Availability

The data from this study are available from the corresponding author upon request.
